# Accuracy of estimated breeding values with genomic information on males, females, or both: an example on broiler chicken

**DOI:** 10.1186/s12711-015-0137-1

**Published:** 2015-07-02

**Authors:** Daniela A. L. Lourenco, Breno O. Fragomeni, Shogo Tsuruta, Ignacio Aguilar, Birgit Zumbach, Rachel J. Hawken, Andres Legarra, Ignacy Misztal

**Affiliations:** Department of Animal and Dairy Science, University of Georgia, Athens, GA 30602 USA; Instituto Nacional de Investigacion Agropecuaria, Las Brujas, 90200 Uruguay; Cobb-Vantress Inc., Siloam Springs, AR 72761 USA; Institut National de la Recherche Agronomique, UMR1388 GenPhySE, 31326 Castanet-Tolosan, France

## Abstract

**Background:**

As more and more genotypes become available, accuracy of genomic evaluations can potentially increase. However, the impact of genotype data on accuracy depends on the structure of the genotyped cohort. For populations such as dairy cattle, the greatest benefit has come from genotyping sires with high accuracy, whereas the benefit due to adding genotypes from cows was smaller. In broiler chicken breeding programs, males have less progeny than dairy bulls, females have more progeny than dairy cows, and most production traits are recorded for both sexes. Consequently, genotyping both sexes in broiler chickens may be more advantageous than in dairy cattle.

**Methods:**

We studied the contribution of genotypes from males and females using a real dataset with genotypes on 15 723 broiler chickens. Genomic evaluations used three training sets that included only males (4648), only females (8100), and both sexes (12 748). Realized accuracies of genomic estimated breeding values (GEBV) were used to evaluate the benefit of including genotypes for different training populations on genomic predictions of young genotyped chickens.

**Results:**

Using genotypes on males, the average increase in accuracy of GEBV over pedigree-based EBV for males and females was 12 and 1 percentage points, respectively. Using female genotypes, this increase was 1 and 18 percentage points, respectively. Using genotypes of both sexes increased accuracies by 19 points for males and 20 points for females. For two traits with similar heritabilities and amounts of information, realized accuracies from cross-validation were lower for the trait that was under strong selection.

**Conclusions:**

Overall, genotyping males and females improves predictions of all young genotyped chickens, regardless of sex. Therefore, when males and females both contribute to genetic progress of the population, genotyping both sexes may be the best option.

## Background

Large amounts of genomic information have accumulated for nearly all livestock species and its use has led to increases in the accuracy of estimated breeding values (EBV) [1]. These increases are mainly due to improved inferences on relationships between individuals and linkage disequilibrium (LD) between quantitative trait loci (QTL) and markers [2]. Higher accuracies are obtained when relationships between animals in the training population are weak and the relationship between the training and validation populations is high [3].

Questions about how the genotyped population should be structured and which animals should be used in the training population are still a matter of debate in all species. In dairy cattle, for example, phenotypes for production traits are collected on females and combined with genotypes of males for successful genomic evaluation. According to Rendel and Robertson [4], genetic progress in a population is a combination of the progress in each of the four paths of selection. In dairy cattle, selection intensities are highest for elite sires of bulls and elite dams of bulls [5] because strong selection pressure can be applied in both these pathways. With genomic selection, very young females can be chosen (e.g., even heifers) as dams of bulls, and elite cows are often genotyped [6]. Although accurate genomic breeding values for females are highly relevant, including female genotypes and phenotypes in the training population resulted in very small increases in the accuracy of evaluation of young dairy bulls [6, 7]. For instance, adding 17 000 female genotypes to 7000 male genotypes increased the accuracy of evaluation of young bulls from 0.70 to 0.72 [8]. This small increase is due to female phenotypes being largely redundant, since these phenotypes are already included in their sire’s information, either explicitly in the form of pseudo-phenotypes, or implicitly, as in the single-step genomic best linear unbiased predictor (ssGBLUP). However, in dairy cattle, genotyping females is useful for intra-herd selection of females [9] and for identifying elite females to produce future sires.

In species such as broiler chickens or pigs, the number of progeny is smaller per male and larger per female than in dairy cattle. Therefore, the impact of female paths on genetic progress is potentially stronger. Also, when phenotypes are recorded on both sexes (e.g., body weight), then not only can female phenotypes contribute to male evaluations but male phenotypes can also contribute to female evaluations. For this reason, genotyping females in these species can make a substantial contribution to accuracy and genetic progress.

Realized accuracies of genetic values can be obtained from the correlation between true and estimated breeding values for the validation population [10]. There are large discrepancies between theoretical accuracy (e.g., by inversion of the coefficient matrix of the mixed model equations) and realized accuracy of EBV in populations under selection, where the latter is noticeably smaller [11]. For genetic values obtained through genomic BLUP methods (GBLUP), the accuracies that are obtained by inversion of the coefficient matrix depend on the assumed allele frequencies [12], although scaling of genomic relationships for compatibility with pedigree relationships [13, 14] reduces this dependency.

The objective of our work was to analyze a commercial broiler chicken population and determine the gains in the accuracy of genomic evaluations on males and females due to the use of genotypes and phenotypes of males, females, or both sexes.

## Methods

### Data

The dataset and variance components used in this study were provided by Cobb-Vantress Inc. (Siloam Springs, AR). The dataset consisted of phenotypes recorded on purebred broiler chickens across four generations for four production traits referred to as T1, T2, T3, and T4; heritabilities for all traits ranged from 0.22 to 0.49, genetic correlations ranged from −0.02 to 0.21 and phenotypic correlations from −0.02 to 0.46 (Table 1). The first trait (T1) was recorded on 196 613 birds, whereas the three other traits (T2, T3 and T4) were recorded on 26, 5, and 26 % of the birds with records for T1, respectively. Traits T1 and T3 were measured on birds at 35 days of age, whereas traits T2 and T4 were measured within a 2-week period after 35 days of age. Multiple measurements for T2 and T4 were combined into a unique record for T2 and for T4. Thus, each trait was analyzed as a single record. The number of birds in the pedigree relationship matrix (**A**) was 198 915.

**Table 1:** Heritabilities (diagonal), genetic correlations (above the diagonal), and phenotypic correlations (below the diagonal) for the four traits

Trait	T1	T2	T3	T4
T1	0.28	−0.05	0.07	0.16
T2	−0.02	0.25	−0.02	0.21
T3	0.46	NA^a^	0.49	−0.19
T4	0.30	0.00	NA	0.22

^a^NA: no pairwise phenotype is available between two traits

Genotypes from the 60 k SNP (single nucleotide polymorphism) panel developed by Groenen et al. [15] were available for 15 723 birds. Quality control of genomic data retained SNPs with call rates greater than 0.9, minor allele frequencies greater than 0.05, and departures from Hardy-Weinberg equilibrium (difference between expected and observed frequency of heterozygous) less than 0.15. Parent-progeny pairs were tested for discrepant homozygous SNPs, and progenies were eliminated when the conflict rate was greater than 1 %. Also, SNPs with an unknown position or located on sex chromosomes were excluded from the analyses. After quality editing, 39 102 autosomal SNPs for 15 723 birds remained for analysis. The genotype file was split by sex and the three genotype datasets (males, females, and both sexes) were used in different analyses. The total numbers of genotyped males and females were 6149 and 9574, respectively and the numbers of genotyped birds with phenotypes for each trait are in Table 2.

**Table 2:** Number of genotyped birds with phenotypes for each trait

Trait	Birds		
	All	Males	Females
T1	12 748	4648	8100
T2	9567	2010	7557
T3	2213	2213	0
T4	9624	2017	7607

The birds that were genotyped were chosen randomly or based on phenotypes, depending on the trait. The dataset available for this study was split into training and validation populations according to date of birth. Thus, 2975 birds born in generation 4 were chosen as validation animals and their phenotypes were removed from the analyses.

### Model and analysis

For traditional pedigree-based and genomic evaluations, the following multiple-trait animal model was used:

$$ {\mathbf{y}}_{\mathrm{t}} = {\mathbf{X}}_{\mathrm{t}}{\mathbf{b}}_{\mathrm{t}} + {\mathbf{Z}}_{\mathrm{t}}{\mathbf{u}}_{\mathrm{t}} + {\mathbf{e}}_{\mathrm{t}}, $$

where t is for traits T1 to T4; **y**, **b**, **u**, and **e** are vectors of phenotypes, fixed effects of sex and generation-hatch interaction, random additive direct genetic effects, and random residuals, respectively; **X** and **Z** are incidence matrices for **b** and **u**, respectively. A vector of random maternal permanent environmental effects was added for T1. Although sex effect was fitted in the model, no sexual dimorphism was considered and the traits on males and females were assumed to have a genetic correlation of 1, which may not always be the case in practice [16].

Genomic evaluations were conducted using ssGBLUP. In this method, the inverse of the numerator relationship matrix (**A**^−1^) in the mixed model equations was replaced by the inverse of the realized relationship matrix (**H**^−1^) [17, 18], which was written as:

$$ {\mathbf{H}}^{\hbox{-} 1} = {\mathbf{A}}^{\hbox{-} 1}+\left[\begin{array}{cc}\hfill \mathbf{0}\hfill & \hfill \mathbf{0}\hfill \\ {}\hfill \mathbf{0}\hfill & \hfill {\left(\upalpha \left(\mathrm{a}+\mathrm{b}\mathbf{G}\right)+\upbeta {\mathbf{A}}_{\mathbf{22}}\right)}^{\hbox{-} 1}\hbox{--}\ {\mathbf{A}}_{\mathbf{22}}^{\hbox{-} 1}\hfill \end{array}\right], $$

where **G** is the genomic relationship matrix that was constructed as in VanRaden [13], using observed allele frequencies; **A**_**22**_^‐ 1^ is the inverse of the pedigree-based relationship matrix for genotyped animals. Weights were assigned for **G** (α = 0.95) and **A**_**22**_ (β = 0.05) to avoid singularity problems [13]. Coefficients a and b were used to match pedigree and genomic relationships [14, 19, 20]. Different **H** matrices were used based on different **G** that contained 2975 birds from the validation population plus one of the three training populations: males (n = 4648), females (n = 8100), and both sexes (n = 12 748).

Traditional and genomic evaluations were computed using the software BLUP90IOD [21, 22]. The convergence criterion was set to 10^−14^ for all evaluations. Variance components used in all analyses were pre-computed by Cobb-Vantress Inc. using the same data and model as presented here.

#### Composition of genomic estimated breeding values from ssGBLUP

We used the composition of genomic estimated breeding values (GEBV) and some general rules to better understand some of our results. In traditional BLUP evaluations, the EBV for an animal i can be expressed as [23]:

$$ {\mathrm{u}}_{\mathrm{i}} = {\mathrm{w}}_1\mathrm{P}{\mathrm{A}}_{\mathrm{i}} + {\mathrm{w}}_2\mathrm{Y}{\mathrm{D}}_{\mathrm{i}} + {\mathrm{w}}_3\mathrm{P}{\mathrm{C}}_{\mathrm{i}}, $$

where PA_i_ is the parent average EBV for animal i, YD_i_ is the yield deviation (phenotype adjusted for the model effects’ solutions other than additive genetic effects and errors) for animal i, and PC_i_ is the progeny contribution for animal i. When both parents are known, the phenotype is available, and each progeny has a known mate, weights w_1_ to w_3_ sum to 1. The decomposition of EBV can be derived by analyzing a row of the mixed model equations for a given animal. More specifically, YD is based on own phenotypic information, PA is the average of the parental EBV, and PC is the sum of the differences between the EBV of any progeny of animal i minus one half of the EBV of each progeny’s dam (or the mate of animal i).

The EBV for an animal i when genomic information is available (GEBV) is [24]:

$$ {\mathrm{u}}_{\mathrm{i}} = {\mathrm{w}}_1\mathrm{P}{\mathrm{A}}_{\mathrm{i}} + {\mathrm{w}}_2\mathrm{Y}{\mathrm{D}}_{\mathrm{i}} + {\mathrm{w}}_3\mathrm{P}{\mathrm{C}}_{\mathrm{i}} + {\mathrm{w}}_4\mathrm{G}{\mathrm{I}}_{\mathrm{i}}, $$

where GI_i_ contains information from genotypes of animal i and all weights sum to 1. According to VanRaden and Wright [24], the weight for GI is:

$$ {\mathrm{w}}_4 = \frac{{\mathrm{g}}^{\mathrm{ii}}\hbox{-} {\mathrm{a}}_{22}^{\mathrm{ii}}}{\mathrm{den}}, $$

where g^ii^ and a_22_^ii^ are the diagonal elements of **G**^−1^ and **A**_**22**_^‐ 1^, respectively; den = 2 + n_r_/α + n_p_/2 + g^ii^ ‐ a_22_^ii^, where n_r_ is the number of records, α is the variance ratio (residual variance over additive genetic variance), and n_p_ is progeny size. Aguilar et al. [17] showed that in ssGBLUP, GI consists of two components:

$$ \mathrm{G}\mathrm{I} = {\mathrm{w}}_{4_1}\mathrm{D}\mathrm{G}\mathrm{V}\hbox{-} {\mathrm{w}}_{4_2}\mathrm{P}\mathrm{P}, $$

where DGV is the portion of prediction due to the genomic information, which comes from **G,** and PP is pedigree prediction that comes from **A**_**22**_. The weights ${\mathrm{w}}_1,\ {\mathrm{w}}_2,\ {\mathrm{w}}_{3,\ }{\mathrm{w}}_{4_1},\ \mathrm{and}\ {\mathrm{w}}_{4_2}$ sum to 1 and values for DGV and PP are equal to:

$$ \mathrm{D}\mathrm{G}{\mathrm{V}}_{\mathrm{i}}=\frac{\hbox{-} {\displaystyle {\sum}_{\mathrm{j},\mathrm{j}\ne \mathrm{i}}{\mathrm{g}}^{\mathrm{i}\mathrm{j}}{\mathrm{u}}^{\mathrm{j}}}}{{\mathrm{g}}^{\mathrm{i}\mathrm{i}}}, $$

$$ \mathrm{P}{\mathrm{P}}_{\mathrm{i}}=\frac{\hbox{-} {\displaystyle {\sum}_{\mathrm{j},\mathrm{j}\ne \mathrm{i}}{\mathrm{a}}_{22}^{\mathrm{i}\mathrm{j}}{\mathrm{u}}^{\mathrm{j}}}}{{\mathrm{a}}_{22}^{\mathrm{i}\mathrm{i}}}, $$

where g^ij^ and a_22_^ij^ are the off-diagonal elements of **G**^−1^ and **A**_**22**_^‐ 1^, respectively; u^j^ is the inverse EBV of animal j.

In general, PP accounts for the part of PA that is explained by DGV; when all animals are genotyped, **A** = **A**_22_, PA and PP cancel out and DGV explains a larger fraction of the GEBV; when a genotyped animal is unrelated to the genotyped population, PP = 0 and DGV explains a smaller portion of the GEBV; when both parents are genotyped, PP will include a large part of PA. The accuracy of DGV differs between animals, depending on how many ancestors of that animal are genotyped, as reported by Mulder et al. [25]. When a genotyped animal has many progeny, w_3_ ≈ 1 and its GEBV is mainly driven by PC; however, genotyping those animals is useful since they are usually included in the training population. When an animal is not genotyped, w_4_ = 0 and predictions can be improved due to improved PA and PC if its relatives are genotyped. When an animal is not genotyped and has no phenotypes and no progeny, the GEBV is driven by PA and, in most cases, only a slight improvement in prediction is achieved based on genotyped relatives [17, 18, 26].

#### Validation

Validation of EBV was based on that proposed by Legarra et al. [10]; predictive ability of traditional and genomic evaluations was defined as the correlation between (G)EBV and trait phenotypes corrected for fixed effects (Y) for birds in the validation population:

$$ \mathrm{r} = \mathrm{c}\mathrm{o}\mathrm{r}\left(\left(\mathrm{G}\right)\mathrm{E}\mathrm{B}\mathrm{V},\mathrm{Y}\right), $$

where (G)EBV can be either EBV or GEBV.

**Fig. 1:**
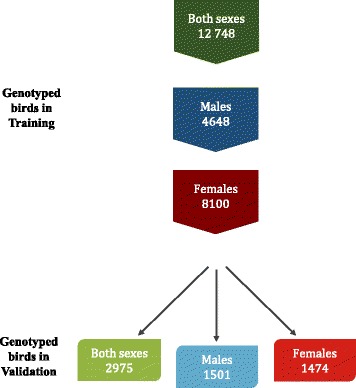
Cross-validation scheme representing birds in training and validation populations

Accuracy, as determined by the correlation between true and predicted breeding values, was calculated as r/h; where h is the square root of heritability [10]. Accuracy was obtained for young birds in the validation population, with and without splitting them into groups according to sex (Fig. 1). Accuracy of GEBV was used to assess the benefit of including genotypes for different sets of birds on predictive ability of birds with the same sex, opposite sexes, and combined; accuracy of EBV was the benchmark used to compare the gain in predictive ability due to genomic information.

#### Correlation between EBV and GEBV

Correlations between EBV and GEBV using genotypes for both sexes were calculated for sires with large (≥500) and small (<50) progeny groups, and for dams with large (≥50) and small (<5) progeny groups to check the importance of progeny size versus genomic information on EBV of proven parents.

## Results and discussion

A summary of the population structure is in Table 3. About half of all parents were genotyped, but in the validation population, 96 % of the parents were genotyped. According to Pszczola et al. [3], animals in the validation population should be closely related to at least some of the animals in the training population in order to obtain more accurate direct genomic values (DGV). In ssGBLUP, the accuracy of GEBV is less affected by genotype structure, because GEBV includes PA (from **A**) and additional pedigree information (from **A**_**22**_), and the latter accounts for a different level of relationship between a given genotyped animal and the genotyped population. In general, additional information due to genomic data is approximately proportional to the square of the difference between pedigree and genomic relationships [27]; the standard deviation of such differences increases for animals that are more related [28–30], but this increase is not equal for all classes of animals since full-sib groups presented greater standard deviation than parent-offspring groups [30], for instance.

**Table 3:** Family structure for all birds and for genotyped birds in the dataset

	Sires	Dams
Number of parents in the dataset	551	5185
Average progeny per parent	357	38
Number of genotyped parents in the training population	276	2752
Average progeny per parent	451	47
Total parents of the validation population	87	762
Number of genotyped parents of the validation population	83	730
Average progeny per parent	34	4

**Fig. 2:**
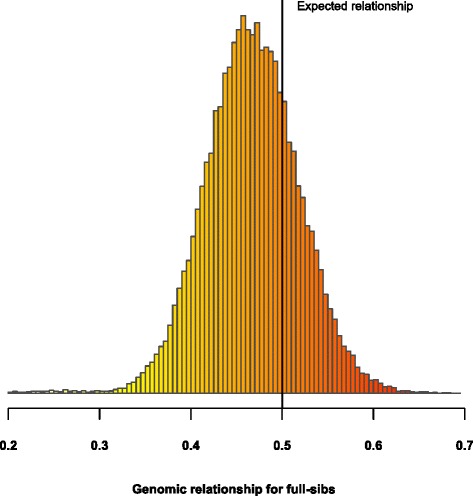
Distribution of genomic relationships for full-sibs among the 15 748 genotyped birds. The expected relationship based on pedigree information is 0.5 (black vertical line)

For quality control, Fig. 2 contains the distribution of genomic relationships for full-sibs. The quality of genomic relationships can also be evaluated for other groups of siblings or by checking all genomic relationships against all pedigree relationships. Broiler chickens have large full-sib families and a greater gain in accuracy is expected from genomic evaluations over traditional evaluations in this case, provided genomic relationships are based on high-quality SNP genotypes. Although the expected relationship among full-sibs in the absence of inbreeding is equal to 0.50, the average (SD) genomic relationship for this dataset was 0.47 (0.05). The standard deviation of 0.05 and the skewed shape agree with theory [12, 23]. However, if the distribution of genomic relationships is not centered on the expected relationship and is long-tailed, genotyping and pedigree errors are present. For the most recent generations, for which stricter quality controls were imposed, such as checking for heritability of gene content as proposed by Forneris et al. [31], the distribution of genomic relationships among full-sibs was nearly normal and centered on 0.5 (data not provided).

### Accuracies and genomic contributions

Correlations between EBV and GEBV were equal to 0.97 and 0.93 for sires with more than 500 and less than 50 progeny, respectively, whereas correlations for dams with more than 50 and less than 5 progeny were equal to 0.89 and 0.88, respectively. Correlations for dams were lower because they have less progeny than males and, as a result, the weight on genotypic information is greater than the weight on PC for dams. For sires, even if there was some re-ranking between EBV and GEBV by including genomic information, the accuracy of the GEBV of sires with many progeny came mostly from PC, because the contribution from other sources was small or null. Although genomic information had a smaller impact on the GEBV of parents with large numbers of progeny, genotyping those birds was helpful to improve predictions from related birds.

**Fig. 3:**
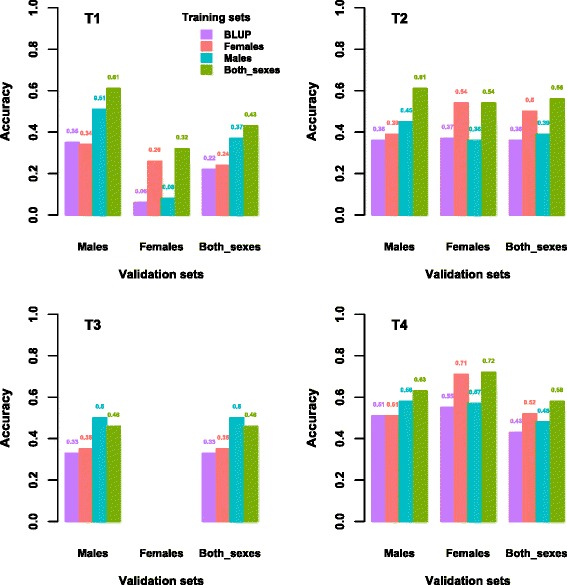
Accuracy of evaluation for all birds, males, and females in the validation population when different sets of genotyped birds were used to construct the **G** matrix. BLUP did not include genotypes and T3 females had no phenotypes

Accuracies for traditional and genomic evaluations are in Fig. 3. Genomic evaluations were derived using three different sets of genotyped birds (only males, only females, and both sexes) in the training population. In all analyses, phenotypes were included for all genotyped animals, except for the youngest chickens that had hatched later in the last generation. In addition, validation sets were also created for young males, young females, and young chickens from both sexes. When the training and validation populations included both sexes, the accuracy of genomic evaluations was always greater (on average, 17 percentage points) than that of traditional evaluations. However, when the genotypes of only one sex for the training population and for both sexes in the validation population were considered, the impact on the accuracy of GEBV differed by trait. For traits T1 and T3, using only female genotypes for the training population resulted in only a slight change in accuracy, whereas using only male genotypes had a much greater impact on accuracy. The opposite was true for traits T2 and T4, for which using only female genotypes had a greater impact than using only male genotypes. These differences can be partially attributed to the number of phenotypes available for genotyped chickens and can be better explained when evaluations of males and females are considered separately.

Traits for which male genotypes had a greater impact (T1 and T3) had either a larger number of phenotypes compared to the other traits, or females had no phenotypes such as T3 (Table 2). For T1, the number of phenotypes on males was 57 % of the number of phenotypes on females, but for T2 and T4 the number of phenotypes on males was roughly 27 % of the number of phenotypes on females. In contrast to using a training population with only males, using genotypes for both sexes improved accuracies for all traits except for T3, for which females had no phenotypes. When males were evaluated, including only female genotypes increased the accuracy only slightly. Also, when females were evaluated, including male genotypes hardly increased accuracies. The same trend was observed by Cooper et al. [32] in a study on the US Holstein population.

Table 4 shows accuracies for pedigree and genomic PA for genotyped and non-genotyped birds. For all traits, accuracies of pedigree PA for non-genotyped birds were greater than for genotyped birds. For non-genotyped birds, the accuracy of genomic PA was very similar to that of pedigree PA for all traits, except for T3, for which the accuracy of genomic PA was greater. For T3, which was measured only on males and for which there were fewer phenotypes than for the other traits, including genomic information improved the accuracy of the GEBV of parents. When the progeny is not genotyped but parents are, realized Mendelian sampling terms from parents to offspring cannot be accurately estimated and gains in accuracy are lower [33]. The gains in accuracy are mainly due to improved accuracy of PA if only the parents are genotyped or also of PC if both parents and progeny are genotyped. Genotyping parents of non-genotyped birds may result in greater benefit for sex-limited traits or when trait recording is limited to a small number of birds. Comparisons between accuracies of genomic PA (Table 4) and genomic EBV (Fig. 3) show that genomic information on genotyped young birds contributes significantly to accuracy of evaluation. Pszczola et al. [33] showed that accuracies of GEBV increased when progenies were genotyped and parents were not, compared to the opposite situation; but still the highest accuracy was achieved when a large portion of the population was genotyped. According to Mulder et al. [25], the number of genotyped ancestor generations affects the accuracy of genomic predictions.

**Table 4:** Accuracy for pedigree and genomic^a^ parent average for genotyped and non-genotyped birds

Status	Parent average	T1	T2	T3	T4
Genotyped	Pedigree	0.22	0.36	0.33	0.43
	Genomic	0.23	0.39	0.34	0.47
Non-genotyped	Pedigree	0.25	0.48	0.36	0.49
	Genomic	0.23	0.47	0.43	0.51

^a^Genomic parent average included genomic information on both sexes; validation was done in both sexes

For males in the validation population, accuracy improved significantly when male genotypes were added to the training population (Fig. 3). Similarly, for females, accuracy improved significantly when female genotypes were added. Consequently, genotypes for a particular sex that are linked to phenotypic information benefit the genotyped birds of that sex. Cooper et al. [32] showed that using only female genotypes in the training population, opposed to using genotypes only on males, was advantageous for predicting the GEBV for cows, and the same was true for bulls; however, adding female genotypes to an already existing training population of bulls resulted in a very small benefit.

In our study, when genotypes of both sexes were included, opposed to using genotypes for one sex, there was an additional increase in accuracy for each sex (Fig. 3). This may be caused by the contribution of males versus females to the population being different in broiler chickens than in dairy cattle, in which males have a much greater impact on the population due to larger progeny groups. Part of this increase is likely due to the use of the ssGBLUP method, which can model phenotypes and genotypes from both sexes when genotypes are not available for the entire population. This method weights the records of males and females and avoids double-counting of phenotypic and pedigree information. It also establishes connections among more animals with independent information (since it avoids double-counting) through genomic relationships, and combines PA and pedigree prediction.

The increase in accuracy from including genotypes of the opposite sex was greater for validation males than for validation females (Fig. 3). This could be due to several factors: (1) the number of genotypes for females was much larger than that for males and consequently more links were established through **H** (as **G** is identical by state) and estimates of DGV and PP were improved; (2) genetic correlations between phenotypes on males and females differ from 1 (our study assumes a correlation of 1); or (3) genomic imprinting is present and thus gene expression depends on the parental origin of the allele [34].

The relative increase in accuracy for females from adding male genotypes was larger for trait T1 than for T4 because T1 had a larger number of male phenotypes (4648) than trait T4 (2017 male phenotypes) (Table 2 and Fig. 3). Since accuracy was computed as the correlation between EBV or GEBV and phenotypes corrected for fixed effects, no accuracy could be computed for T3 for females because this trait was only recorded for males. Therefore, there was no improvement in accuracy of GEBV from adding female genotypes for T3. In fact, the accuracy deteriorated slightly from 0.50 to 0.46, although adding genotypes is not expected to decrease accuracy if the model is correct, the genomic information is accurate, and all selection is accounted for. Thus, the observed decrease in accuracy could be due to modeling issues, e.g., insufficient modeling of factors associated with T3, structure of the validation population, unaccounted selection, or sexual dimorphism [35].

Our study ignored sexual dimorphism [16, 35, 36] because genetic correlations between sexes were assumed to be equal to 1. If this assumption does not hold, realized accuracies could be higher with proper modeling. Follow-up research is required to evaluate the change in ranking for animals evaluated for different traits when sexual dimorphism is accounted for and genomic information is available.

### Realized accuracy and accuracy from the inverse of the coefficient matrix of the mixed model equations

**Fig. 4:**
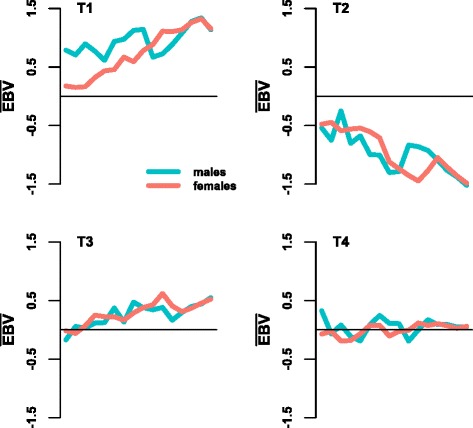
Genetic trends based on traditional EBV for all traits for genotyped males and females. Trends are shown over generations and were obtained from a multi-trait model of all four traits

In spite of a large number of genotyped birds, the overall accuracies obtained for the dataset used in this study were below expectations. The maximum theoretical accuracy with PA is 0.71; however, the average accuracy was only 0.35 for BLUP and 0.54 for ssGBLUP with birds from both sexes in the training population. VanRaden et al. [1] obtained, respectively, 0.44 and 0.60 for dairy bulls. Realized accuracies in selected populations are smaller than accuracies by inversion of the coefficient matrix of the mixed model equations, if selection is not accounted for [1, 11], with lower realized accuracies under stronger direct selection [37]. In this study, traits T2 and T4 had similar numbers of phenotypes (within a gender) and genotypes, and similar heritabilities. Yet, average accuracies of EBV were up to 48 % higher for T4 than for T2, with differences being larger for females. This suggests that differential selection pressure is placed on these two traits. Indeed, T2 was strongly selected for, while genetic trends for T4 showed no selection pressure in any direction (Fig. 4). While accuracies of EBV and GEBV for a weakly selected trait such as T4 were higher for females than for males, accuracies for females were slightly lower than for males for T2 and much lower for T1. Parents of the validation population were selected in a generation in which the selection pressure for females was higher than for males for T1 and T2. The very low accuracy for females for T1, especially with BLUP, was due to strong phenotypic preselection of females based on T1; in case of extreme selection, the realized accuracy tends towards zero. When selection takes place, cross-validation accuracy differs from accuracy obtained by inversion of the coefficient matrix of the mixed model equations, and adjusting the latter is notoriously difficult since it would require selection differentials; however, selection is a multiple trait and possibly multistage process but the exact process is unknown, and selection intensity varies depending on the selection pathway [11].

## Conclusions

Accuracies in genomic selection depend on the number, distribution, and contributions of genotypes and phenotypes to the genomic evaluation. Contrary to what has been reported for dairy cattle, in this chicken population, the gain in accuracy of GEBV for young genotyped animals was higher when the training population included genotypes for both males and females. We also observed that when the training population has only animals from one sex, the greatest benefit is for young genotyped animals from the same sex. However, when both sexes are genotyped, the amount of genomic information increases greatly and accuracy of GEBV also increases. Thus, genotyping both sexes may be a suitable option in species and production systems for which not only males but also females have a high reproductive impact. For highly selected traits, realized accuracy of GEBV is smaller because it accounts for selection.

## References

[1] VanRaden PM, VanTassel CP, Wiggans GR, Sonstegard TS, Schnabel RD, Taylor JF (2009). Invited review: reliability of genomic predictions for North American Holstein bulls. J Dairy Sci.

[2] Daetwyler HD, Kemper KE, van der Werf JH, Hayes BJ (2012). Components of the accuracy of genomic prediction in a multi-breed sheep population. J Anim Sci.

[3] Pszczola M, Strabel T, Mulder HA, Calus MPL (2012). Reliability of direct genomic values for animals with different relationships within and to the reference population. J Dairy Sci.

[4] Rendel JM, Robertson A (1950). Estimation of genetic gain in milk yield by selection in a closed herd of dairy cattle. J Genet.

[5] Schaeffer LR (2006). Strategy for applying genome-wide selection in dairy cattle. J Anim Breed Genet.

[6] Wiggans GR, Cooper TA, VanRaden PM, Cole JB (2011). Technical note: adjustment of traditional cow evaluations to improve accuracy of genomic predictions. J Dairy Sci.

[7] Tsuruta S, Misztal I, Lawlor TJ (2013). Short communication: genomic evaluations of final score for US Holsteins benefit from the inclusion of genotypes on cows. J Dairy Sci.

[8] Harris BL, Winkelman AM, Johnson DL (2013). Impact of including a large number of female genotypes on genomic selection. Interbull Bull.

[9] Di Croce FA, Osterstock JB, Weigel DJ, Lormore MJ (2014). Gains in reliability with genomic information in US commercial Holstein heifers [abstract]. J Dairy Sci.

[10] Legarra A, Granie CR, Manfredi E, Elsen JM (2008). Performance of genomic selection in mice. Genetics.

[11] Bijma P (2012). Accuracies of estimated breeding values from ordinary genetic evaluations do not reflect the correlation between true and estimated breeding values in selected populations. J Anim Breed Genet.

[12] Stranden I, Christensen OF (2011). Allele coding in genomic evaluation. Genet Sel Evol.

[13] VanRaden PM (2008). Efficient methods to compute genomic predictions. J Dairy Sci.

[14] Vitezica ZG, Aguilar I, Misztal I, Legarra A (2011). Bias in genomic predictions for populations under selection. Genet Res (Camb).

[15] Groenen MA, Megens HJ, Zare Y, Warren WC, Hillier LW, Crooijmans RP (2011). The development and characterization of a 60 K SNP chip for chicken. BMC Genomics.

[16] Closter AM, van As P, Elferink MG, Crooijmanns RPMA, Groenen MAM, Vereijken ALJ (2012). Genetic correlation between heart ratio and body weight as a function of ascites frequency in broilers split up into sex and health status. Poult Sci.

[17] Aguilar I, Misztal I, Johnson DL, Legarra A, Tsuruta S, Lawlor TJ (2010). Hot topic: a unified approach to utilize phenotypic, full pedigree, and genomic information for genetic evaluation of Holstein final score. J Dairy Sci.

[18] Christensen OF, Lund MS (2010). Genomic prediction when some animals are not genotyped. Genet Sel Evol.

[19] Chen CY, Misztal I, Aguilar I, Legarra A, Muir WM (2011). Effect of different genomic relationship matrices on accuracy and scale. J Anim Sci.

[20] Christensen OF (2012). Compatibility of pedigree-based and marker-based relationship matrices for single-step genetic evaluation. Genet Sel Evol.

[21] Aguilar I, Misztal I, Legarra A, Tsuruta S (2011). Efficient computation of the genomic relationship matrix and other matrices used in single-step evaluation. J Anim Breed Genet.

[22] Tsuruta S, Misztal I, Strandén I (2001). Use of the preconditioned conjugate gradient algorithm as a generic solver for mixed-model equations in animal breeding applications. J Anim Sci.

[23] VanRaden PM, Wiggans GR (1991). Deviation, calculation, and use of national animal model information. J Dairy Sci.

[24] VanRaden PM, Wright JR (2013). Measuring genomic pre-selection in theory and in practice. Interbull Bull.

[25] Mulder HA, Calus MPL, Druet T, Schrooten C (2012). Imputation of genotypes with low-density chips and its effect on reliability of direct genomic values in Dutch Holstein cattle. J Dairy Sci.

[26] Legarra A, Aguilar I, Misztal I (2009). A relationship matrix including full pedigree and genomic information. J Dairy Sci.

[27] Misztal I, Tsuruta S, Aguilar I, Legarra A, VanRaden PM, Lawlor TJ (2013). Methods to approximate reliabilities in single-step genomic evaluation. J Dairy Sci.

[28] Garcia-Cortes LA, Legarra A, Chevalet C, Toro MA (2013). Variance and covariance of actual relationships between relatives at one locus. PLoS One.

[29] Hill WG, Weir BS (2011). Variation in actual relationship as a consequence of Mendelian sampling and linkage. Genet Res (Camb).

[30] Wang H, Misztal I, Legarra A (2014). Differences between genomic-based and pedigree-based relationships in a chicken population, as a function of quality control and pedigree links among individuals. J Anim Breed Genet.

[31] Forneris NS, Legarra A, Vitezica ZG, Tsuruta S, Aguilar I, Misztal I (2015). Quality control of genotypes using heritability estimates of gene content at the marker. Genetics.

[32] Cooper TA, Wiggans GR, VanRaden PM (2015). Short Communication: analysis of genomic predictor population for Holstein dairy cattle in the United States–effects of sex and age. J Dairy Sci.

[33] Pszczola M, Strabel T, van Arendonk JAM, Calus M (2012). The impact of genotyping different groups of animals on accuracy when moving from traditional to genomic selection. J Dairy Sci.

[34] de Koning DJ, Rattink AP, Harlizius B, van Arendonk JA, Brascamp EW, Groenen MA (2000). Genome-wide scan for body composition in pigs reveals important role of imprinting. Proc Natl Acad Sci USA.

[35] Mignon-Gasteau S, Beaumont C, Poivey JP, Rochambeau H (1998). Estimation of the genetic parameters of sexual dimorphism of body weight in’label’ chickens and Muscovy ducks. Genet Sel Evol.

[36] Maniatis G, Demiris N, Kranis A, Banos G, Kominakis A (2013). Genetic analysis of sexual dimorphism of body weight in broilers. J Appl Genet.

[37] Edel C, Neuner S, Emmerling R, Gotz KU (2012). A note on ‘forward prediction’ to access precision and bias of genomic predictions. Interbull Bull.

